# The draft genome sequence of the Japanese rhinoceros beetle *Trypoxylus dichotomu*s *septentrionalis* towards an understanding of horn formation

**DOI:** 10.1038/s41598-023-35246-w

**Published:** 2023-05-30

**Authors:** Shinichi Morita, Tomoko F. Shibata, Tomoaki Nishiyama, Yuuki Kobayashi, Katsushi Yamaguchi, Kouhei Toga, Takahiro Ohde, Hiroki Gotoh, Takaaki Kojima, Jesse N. Weber, Marco Salvemini, Takahiro Bino, Mutsuki Mase, Moe Nakata, Tomoko Mori, Shogo Mori, Richard Cornette, Kazuki Sakura, Laura C. Lavine, Douglas J. Emlen, Teruyuki Niimi, Shuji Shigenobu

**Affiliations:** 1 Division of Evolutionary Developmental Biology, National Institute for Basic Biology, Okazaki, Japan; 2 Department of Basic Biology, School of Life Science, The Graduate University for Advanced Studies, SOKENDAI, Okazaki, Japan; 3 Division of Evolutionary Biology, National Institute for Basic Biology, Okazaki, Japan; 4 Division of Integrated Omics Research, Research Center for Experimental Modeling of Human Disease, Kanazawa University, Kanazawa, Japan; 5 Laboratory of Evolutionary Genomics, National Institute for Basic Biology, Okazaki, Japan; 6 Trans-Omics Facility, National Institute for Basic Biology, Okazaki, Japan; 7 Laboratory of Sericulture and Entomoresources, Graduate School of Bioagricultural Sciences, Nagoya University, Nagoya, Japan; 8 URA Division, Office of Research and Academia-Government-Community Collaboration, Hiroshima University, Hiroshima, Japan; 9 Department of Applied Biosciences, Graduate School of Agriculture, Kyoto University, Kyoto, Japan; 10 Department of Biological Science, Faculty of Science, Shizuoka University, Shizuoka, Japan; 11 Laboratory of Molecular Biotechnology, Graduate School of Bioagricultural Sciences, Nagoya University, Nagoya, Japan; 12 Department of Agrobiological Resources, Faculty of Agriculture, Meijo University, Nagoya, Japan; 13 Department of Integrative Biology, University of Wisconsin-Madison, Madison, WI USA; 14 Department of Biology, University of Naples Federico II, Naples, Italy; 15 Institute of Agrobiological Sciences, National Agriculture and Food Research Organization, Tsukuba, Japan; 16 Department of Entomology, Washington State University, Pullman, WA USA; 17 Division of Biological Sciences, The University of Montana, Missoula, MT USA

**Keywords:** Genomics, Entomology, Evolutionary developmental biology

## Abstract

The Japanese rhinoceros beetle *Trypoxylus dichotomus* is a giant beetle with distinctive exaggerated horns present on the head and prothoracic regions of the male. *T. dichotomus* has been used as a research model in various fields such as evolutionary developmental biology, ecology, ethology, biomimetics, and drug discovery. In this study, de novo assembly of 615 Mb, representing 80% of the genome estimated by flow cytometry, was obtained using the 10 × Chromium platform. The scaffold N50 length of the genome assembly was 8.02 Mb, with repetitive elements predicted to comprise 49.5% of the assembly. In total, 23,987 protein-coding genes were predicted in the genome. In addition, de novo assembly of the mitochondrial genome yielded a contig of 20,217 bp. We also analyzed the transcriptome by generating 16 RNA-seq libraries from a variety of tissues of both sexes and developmental stages, which allowed us to identify 13 co-expressed gene modules. We focused on the genes related to horn formation and obtained new insights into the evolution of the gene repertoire and sexual dimorphism as exemplified by the sex-specific splicing pattern of the *doublesex* gene. This genomic information will be an excellent resource for further functional and evolutionary analyses, including the evolutionary origin and genetic regulation of beetle horns and the molecular mechanisms underlying sexual dimorphism.

## Introduction

Beetles (Insecta: Coleoptera) are the largest order not only among insects but among all animals, and they are regarded as one of the most successful animal groups^1^. Beetles exhibit extraordinary morphological, ecological, and behavioral diversity^2^ and have been used as models to study ecological and evolutionary biology for centuries. For example, when Darwin introduced the term "sexual selection," the beetle horn was featured as a typical example^3^.

**Figure 1:**
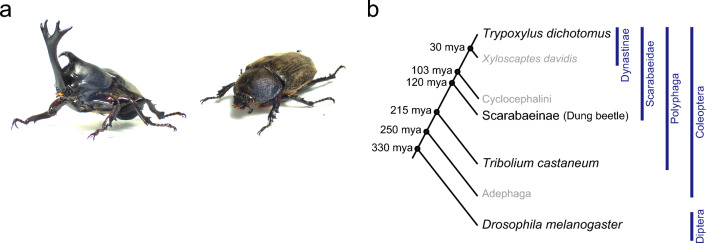
Photograph and phylogenetic context of the Japanese rhinoceros beetle, *Trypoxylus dichotomus*. (**a**) Photograph of an adult male (left) and an adult female (right) of *T. dichotomus.* (**b**) A phylogenetic tree of beetles depicting the phylogenetic relationship between *T. dichotomus* and related beetles with *D. melanogaste*r as an outgroup. Estimated divergence dates (mya: million years ago) are based on Hunt et al., Ahrens et al., Misof et al., Mckenna et al. and Jin et al.^1,5–8^.

*Trypoxylus dichotomus,* commonly known as the Japanese rhinoceros beetle, is a giant beetle reaching up to 91.7 mm in length (Fig. 1a). First described by Linnaeus^4^, *T. dichotomus* belongs to the Scarabaeidae, Dynastini, Trypoxylus (Fig. 1b). The genus *Trypoxylus* is most closely related to the genus *Xyloscaptes* (Fig. 1b)^1,5–8^. *T. dichotomus* inhabits East Asia including Japan, China, Tibet, Taiwan, the Korean peninsula, Northeastern India, Thailand, Vietnam, Laos, and Myanmar. *T. dichotomus* is characterized by the set of exaggerated horns present in the head and prothoracic regions of males, while females have no horns. The horn of the head region (head horn) can extend to more than 1/3 of the male’s body length and is bifurcated twice at the distal tip. The horn of the prothoracic region (thoracic horn) is shorter and bifurcated once at the distal tip. Both horns are strongly sexually dimorphic, and are used as weapons in combat between males over feeding territories visited by females^9^. Battles take place on the trunks of host trees, with males inserting their head horn under the prothorax of an opponent and attempting to scoop the rival off of the tree. Biomechanical studies of horn morphology indicate that the shape of the horn, particularly the triangular cross-section of the base of the horn, resists buckling when twisted and is well suited to the nature of the battles in this species^10^.

*T. dichotomus* is a univoltine insect. Larvae feed and grow on humus soil or decaying wood from autumn to spring. During the prepupal stage, the larvae form a pupal chamber using a mixture of fecal pellets and humus to pupate below ground. In early summer, the adults appear in broad-leaved forests to ingest sap, and males and females mate on trees near feeding sites. After mating, the female lays the eggs in the humus. Adults have a maximum lifespan of about three months and are unable to overwinter.

*T. dichotomus* is an emerging model insect with many advantages. First, it is easy to obtain and the breeding/culturing system in the laboratory has been well developed. Larvae are easily maintained in the laboratory and can be stored at low temperatures to delay the onset of pupation, facilitating their use for research throughout the year^11^. For the critical period from prepupa to adult, when horn formation occurs, a soil-free breeding system has been established that permits non-invasive continuous monitoring and the precise staging of horn development^12^. Second, an RNA interference (RNAi) technique has been established in *T. dichotomus*, which allows functional assessment of genes of interest^11–17^. Larval RNAi is performed by injecting double-stranded RNA (dsRNA) into the 1st thoracic segment (T1 segment) of the last instar larva just before the prepupal stage. Larval RNAi is so efficient that researchers can carry out large-scale RNAi screens against candidate genes, as proved by our successful identification of genes related to horn formation^11–16^. Third, the large body size of *T. dichotomus* has advantages in efficient sampling for various experiments, which could facilitate biochemical assays and molecular analyses including next-generation sequencing (NGS)^15,18^. For example, sufficient amounts of RNA and DNA required for NGS studies can be obtained from each tissue of a single individual alone as shown in this paper.

Studies of *T. dichotomus* have increased rapidly in a wide variety of research fields due to the utility of this species as a model system. For example, in developmental biology the mechanisms of *T. dichotomus* horn formation have been described in depth^19^. The molecular pathways underlying sexual dimorphism of the exaggerated horns have been intensively studied^11,12^, and numerous genes involved in horn formation have been identified by comprehensive transcriptome analyses^15^. In addition, beetle horns are considered to be an evolutionary novelty because there is no obvious homologous structure in the ancestral species^20^, and *T. dichotomus* horns have proven to be an excellent model for exploring how novel morphological structures arise.

The length of *T. dichotomus* horns exhibits a highly sensitive response to the nutritional condition of larvae^21^. Many exaggerated ornaments and weapons of sexual selection exhibit such nutrition- or condition-dependent expression. This plasticity is considered to be an integral component of their function as a reliable signal of the quality of a male. The horns of *T. dichotomus* were the first sexually-selected structure of any animal species to have these underlying mechanisms of conditional expression explored at a developmental or genetic level^13^. The resulting variation in male horn length is mildly polyphenic, yielding major and minor males^9,22^, and ethological studies revealed that males tap each other with their head horns before direct combat^23^, to assess the size of their competitors and avoid unnecessary fighting^24^. Less competitive minor males have alternative reproductive strategies to spend more time mating with females^25^ and appear at feeding sites earlier than major males to avoid fighting and encounter females^26,27^.

The large size and extraordinary morphology of *T. dichotomus* has inspired several biomimetic studies. For example, the mechanical properties of elytra (fore wings)^28–31^ and the aerodynamic mechanisms^32^ of *T. dichotomus* flight have been studied and applied to industrial uses. Furthermore, the high degradation efficiency of *T. dichotomus* for lignocellulose, which is difficult to degrade, is expected to be effectively utilized in the biotreatment of plant biomass^33–36^. In addition, an anti-bacterial peptide^37–43^ and a molecule with anti-prion activity^44^ were discovered from *T. dichotomus* and medical applications of these molecules are expected. Thus, *T. dichotomus* has been actively used in various research fields, including evolutionary developmental biology, ecology, ethology, biomimetics, and drug discovery.

Despite the excellent properties of this beetle as a research model, limited genetic information has been available for *T. dichotomus* until recently, despite a growing number of NGS studies including genomic and transcriptomic analyses being reported^15,18,45,46^. In the present study, we present the draft genome of *T. dichotomus septentrionalis,* along with functional annotation and gene expression data. In addition, we compare the gene repertoire of *T. dichotomus* with those of three other insects. We focus especially on the enlarged male horns of this species, aiming to gain a deeper understanding of the genetic basis of horn formation and the evolution of these genes. This genomic information provides a crucial foundation for future studies using *T. dichotomus* as well as the comparative genomics of insects.

## Materials and methods

### Insects

We purchased *T. dichotomus septentrionalis* larvae from Loiinne (Gunma, Japan). The sex of last (third) instar larvae were determined according to previously described methods^11^, and the larvae were then individually fed on humus in plastic containers, and kept at 10 °C until use. For tissue sampling, larvae were moved to room temperature for a minimum of 10 days and reared at 28 °C.

### Genome sequencing and de novo assembly

Male leg primordia derived from a single individual at 72 h after pupal-chamber formation, which is before cuticle pigmentation and sclerotization, were dissected out from prepupa in 0.75% sodium chloride solution, and used for genome analysis.

To prepare high molecular weight (HMW) DNA of the beetle, 184.5 mg of the frozen tissue was transferred to a mortar and gently ground into a fine powder with liquid nitrogen. Frozen powdery QIAGEN G2 buffer (Qiagen, Netherlands, Cat #1014636), which was generated by spraying the buffer into liquid nitrogen in a glass beaker, was added to the sample and blended quickly. Letting the mixture thaw in a tube, RNase A (QIAGEN, Cat #19101) and Proteinase K (QIAGEN, Cat #1019499) were added, and the sample was incubated at room temperature for 2.5 h without agitation. The sample was centrifuged at 5000 × g for 30 min and the supernatant was subjected to DNA extraction with a QIAGEN Genomic-tip 100/G column (Cat # 10243). The genomic DNA was eluted with 5 ml of Buffer QF, and then purified and concentrated using 0.5-fold volume of Agencourt AMPure XP magnetic beads (Beckman Coulter, Brea, CA, USA, Cat # A63881) yielding 12.2 µg of DNA (174.0 ng/µl). The DNA was size-fractioned by using SAGE HLS (Sage Science, Beverly, MA, USA, Cat #HLS0001), and the fraction with the highest molecular size was collected. The size distribution of the HMW DNA was evaluated by pulsed-field-gel-electrophoresis (PFGE). In brief, 20 ng of HMW DNA was run on a 1% agarose gel (Seakem Gold Agarose, Lonza, Rockland, ME, USA, Cat #50150) in 0.5xTBE with the BioRad CHEF Mapper system (BioRad, Hercules, CA, USA, Cat #M1703650) for 15 h, and the gel was stained with SYBR Gold dye (Thermo Fisher Scientific, Waltham, MA, USA, Cat #11494). Lambda ladder, *Saccharomyces cerevisiae* genome, and 5 kbp ladder (BioRad, Cat #170–3624) were used as standards. These results demonstrated the HMW DNA had an approximate mean size of 50–80 kbp, while shorter DNA fragments (< ~ 10 kbp) were efficiently removed (Fig. S1). DNA concentration was measured with a Qubit fluorometer (Thermo Fisher Scientific, Cat #Q32866) using the Qubit™ dsDNA BR Assay Kit (Thermo Fisher Scientific, Cat #Q32850).

We constructed a 10 × Genomics Chromium linked-read library using 0.68 ng of the HMW DNA extracted as described above with a Chromium Genome Library Kit & Gel Bead Kit v2 (10 × Genomics, San Francisco, CA, USA, Cat #120258) following the manufacturer's protocol. The generated library was sequenced on a HiSeqX (IIlumina, San Diego, CA, USA) at Macrogen Japan Corp (Tokyo, Japan). The obtained Illumina reads were assembled using the Supernova assembler (ver. 2.1.0)^47^ with a parameter of maxreads = 265000000.

We assembled the mitochondrial genome of *T. dichotomus* separately from a paired-end library. Genomic DNA was extracted from the leg primordia using the QIAGEN Genomic-Tip. A paired-end library was prepared with the TruSeq DNA PCR-Free Library Preparation Kit (Illumina) from 1 µg of the genomic DNA. The library was sequenced using the Illumina HiSeqX system with 2 × 150 bp paired-end sequencing protocol at Macrogen Japan Corp (Tokyo, Japan), where 182,102,568 read-pairs were produced. The mitochondrial genome was assembled using a subset of the raw reads (1 M reads, 302 Mb) by NovoPlasty (v4.3.1)^48^ with a parameter of K-mer = 33, using a partial sequence of 16S rRNA of *T. dichotomus* (GenBank accession: AB178318.1) as the seed sequence. Our de novo assembly yielded two contigs, Contig1 (20,217 bp) and Contig2 (1163 bp). A manual inspection revealed that Contig2 was a repetitive sequence included in Contig1 and Contig1 corresponds to the mitochondrial genome. Note that the repetitive regions were not fully resolved by the de novo assembly from Illumina short reads and it is likely the contig, in fact, contains more units of the repeats. The assembled contig was annotated using MITOS2 webserver (http://mitos2.bioinf.uni-leipzig.de/index.py)^49^ with the invertebrate mitochondrial genetic code. The circular map of the mitochondrial genome was drawn using OrganellarGenomeDRAW^50^.

### Genome annotation

Repeat annotation: We constructed the model of repetitive elements in the genome of *T. dichotomus*, using RepeatModeler (ver. open-1.0.8)^51^ with the NCBI search engine. The repeat content was identified with RepeatMasker (ver. open-4.0.6)^52^ using the result of RepeatModeler as a custom library. Other options were not specified.

Gene prediction: We annotated the *T. dichotomu*s genome for protein-coding genes using RNA-seq data. We generated 16 libraries covering 6 tissues and 6 developmental time points from early embryogenesis to postembryonic stages (see *RNA-Seq analysis* section below). The Illumina RNA-seq reads were aligned against a hard-masked genome in which genomic repetitive elements were substituted by ‘N’, using HISAT2 (ver. 2.1.0)^53^ with default parameters. The BRAKER2 (ver. 2.1.5)^54^ pipeline was used to predict protein-coding genes based on the RNA-seq alignments.

Gene annotation: All predicted protein-coding genes were compared with the NCBI non-redundant protein database (nr DB, release November 2020) using the blastp command of diamond software (ver. 2.0.5)^55^ with a threshold of e-value < 1.0e−5. We used InterProScan (ver. 5.48)^56^ to query the predicted coding regions for known functional domains and assign Gene Ontology (GO) terms to the proteins. We also used the eggNOG-mapper pipeline (ver. 2.0.1b)^57^ for functional annotation (including GO term assignments) of the predicted genes based on the orthology information.

BUSCO (ver. 4.0.6)^58^ was used in quantitative measuring for the assessment of genome assembly, using insecta_odb10 (1367 total orthogroups) as the lineage input. A genome browser was built using JBrowse2 web (ver. 1.5.1)^59^ and is available at http://www.insect.nibb.info/trydi/.

### Ortholog analysis

We used the OrthoFinder (ver. 2.3.11)^60^ to generate clusters of orthologous and paralogous gene families. Public gene datasets of *Onthophagus taurus* (RefSeq accession No. GCF_000648695), *Tribolium castaneum* (UniProt accession No. UP000007266), and *Drosophila melanogaster* (UniProt accession No. UP000000803) were used as references.

### RNA-Seq analysis

In total, 16 samples (egg, non-sexed embryos from five stages, ovary, testis, and Malpighian tubule, hindgut, brain and fat body of males and females, respectively (Table S1)) were used for RNA-seq analysis. Eggs, ovaries, and testes were dissected out from adult insects in 0.75% sodium chloride solution. Malpighian tubule, hindgut, brain and fat body were dissected out from third instar larvae in 0.75% sodium chloride solution. These samples were frozen in liquid nitrogen and stored at − 80 °C until use.

Total RNA was extracted from each tissue sample, except for the fat body sample, using the RNeasy Micro Kit (QIAGEN, Cat # 74004) according to the manufacturer's instructions. Total RNA of the fat bodies was extracted using TRIzol Reagent (Thermo Fisher Scientific, Cat #15596-026) according to the manufacturer's instructions. These total RNAs were treated with DNase using RNase-Free DNase Set (QIAGEN, Cat #79254), from which Illumina sequencing libraries were prepared using the TruSeq Stranded mRNA library prep kit (Illumina) following the manufacturer's instructions. The libraries were multiplexed and sequenced using the Illumina HiSeq1500 system with 2 × 101 bp paired-end sequencing protocol at the Functional Genomics Facility of National Institute for Basic Biology.

*T. dichotomus* RNA-Seq reads generated from these 16 libraries were adapter-trimmed using Trim Galore! (ver. 0.5.0)^61^ and cutadapt (ver. 1.18)^62^. The cleaned RNA-Seq reads were mapped to the *T. dichotomus* genome assembly using HISAT2 (ver. 2.1.0)^53^ with default parameters. For quantification of gene expression, StringTie (ver. 2.1.3)^63^ and prepDE.py (http://ccb.jhu.edu/software/stringtie/dl/prepDE.py) with a GTF file of the BRAKER2 gene prediction were used to generate the count matrix. The count data were normalized by the trimmed mean M values (TMM) method available in the edgeR library (ver. 3.32.1)^64–66^. To visualize profiles of gene expression, a multidimensional scaling (MDS) plot was generated using the edgeR software package.

To detect modules of co-expressed genes from the transcriptome data, Weighted Gene Correlation Network Analysis (WGCNA) was applied^67^. Normalized count data were used for this analysis implemented in the WGCNA library (version 1.70)^68^. Genes expressed at low levels, and genes with low expression variance across the libraries, were filtered out; the 6666 surviving gene models were used in the WGCNA analysis. A signed network was constructed in WGCNA with specific parameter settings of power = 8, networkType = "signed", TOMType = "unsigned", minModuleSize = 30.

### Genome size estimation using flow cytometry

Flow cytometry estimates were made by quantification of fluorescence from propidium iodide (PI) stained nuclei extracted from primordia of horns, legs or wings of male pupae with the fruitfly, *D. melanogaster* (1C = 173.3 Mbp) as an internal standard. Samples were added to 1 mL of PBS and homogenized with BioMasher II (nippi, Tokyo, Japan, cat# 320102), and then 1 µl of 10% Triton-X (SIGMA-ALDRICH, St Louis, MO, USA, cat# 93443) and 4 µl of 100 mg/ml RNase A (QIAGEN, cat# 19101) were added to the homogenate. The resultant solution was passed through a 30 µm CellTrics filter (Sysmex Partec, Görlitz, Germany, cat# BP486257) and stained with 10 µg/ml PI (Sony Biotechnology, San Jose, CA, USA, cat# 2706505). The nuclei were analyzed on a Cell Sorter SH800 (SONY Biotechnology) for three replicates.

### Genome size estimation from reads of shotgun sequencing

Genomic DNA was extracted from the leg primordia using the QIAGEN Genomic-Tip (Cat # 10243). A paired-end library was prepared with the TruSeq DNA PCR-Free LT Sample Preparation Kit (Illumina, #FC-121-3001) from 1 µg of the genomic DNA. The library was sequenced using the Illumina HiSeqX system with 2 × 150 bp paired-end sequencing protocol at Macrogen Japan Corp (Tokyo, Japan), where 182,102,568 read-pairs were produced. To estimate the genome size of *T. dichotomus,* we analyzed the distribution of k-mers from the Illumina reads using Jellyfish (ver. 2.2.10)^69^ and GenomeScope 2.0 (git commit id: fdeb891)^70^. The distribution of k-mers of size was analyzed with three different k-mer sizes, 21, 31 and 41.

### Whole genome alignment

We compared our *T. dichotomus* assembly with another published assembly derived from the Chinese population, as reported by Wang et al.^46^. The assembly of *T. dichotomus* isolate ZJPA, in FASTA format, was downloaded from the publication’s supporting data website on GigaScience (http://gigadb.org/dataset/102226). Whole genome alignment of the two assemblies was conducted using unimap ver. 0.1-r46 (https://github.com/lh3/unimap), a variant of minimap2^71^ optimized for assembly-to-reference alignment, with the following parameters: -x asm10. The generated alignment result, in PAF format, was then analyzed using paftools, a utility program included in the minimap2 distribution, to obtain information on structural variations. Dot plot visualization was conducted with D-Genies^72^.

## Results and discussion

### Genome size estimate

Using flow cytometry and the fruitfly *D. melanogaster* as reference, we determined that the haploid genome size of *T. dichotomus* is 773.1 ± 24.6 Mb. In addition, we estimated the *T. dichotomus* genome size from the distribution of k-mers from Illumina reads of shotgun sequences. The distribution of k-mers of size 21, 31 and 41 resulted in an estimated haploid genome size of 710–764 Mb. The small discrepancy between k-mer and cytometry-based estimates may be caused by the repetitive elements (see below), which can affect k-mer estimates.

### Genome assembly and evaluation

Genome sequencing of *T. dichotomus* was performed with genomic DNA isolated from leg primordia of a single male prepupa collected in Gunma, Japan. We prepared high molecular weight (HMW) DNA from the beetle sample with an approximate mean size of 50–80 kbp. The HMW genome DNA (0.68 ng) was subjected to the linked-read whole-genome sequencing library construction on the 10 × Chromium platform. A draft genome was assembled with Supernova^47^ using 265.02 M Illumina reads (57.0 × coverage). The haploid genome assembly was named TdicSN1.0 and used for downstream analysis.

The final assembly TdicSN1.0 consists of 15,609 scaffolds with an N50 of 8.02 Mb and a total size of 615 Mb (Table 1), covering 80% of the genome. We evaluated the completeness of the assembly by using the benchmarking universal single-copy orthologs (BUSCO)^73^. The BUSCO analysis showed that our *T. dichotomus* genome assembly has high coverage of coding regions, capturing 99.4% (98.9% complete, 0.5% fragmented) from the Insecta dataset (version 4.0.6; n = 1367) (Table 2). The score is comparable to the genome of the model beetle *T. castaneum*^74^.

**Table 1:** Summary of *T. dichotomus* genome assembly.

Genome	Assembly size*	615,027,545
	No. scaffolds	15,609
	Scaffold N50	8,021,426
	Longest scaffold	25,115,014
	GC%	34.8
	No. Ns	20.0 Mb
Annotation	No. coding genes	23,987
	Repeat content	305 Mb (49.5%)

*Genome size estimated with flow cytometry or k-mer distibution is 710–773 Mb. (See main texts).

**Table 2:** BUSCO analysis of the genome assembly of *T. dichotomus* using Insecta gene set.

Database	insecta_odb10	
Complete BUSCOs	1352	98.9%
Complete and single-copy BUSCOs	1335	97.7%
Complete and duplicated BUSCOs	17	1.2%
Fragmented BUSCOs	7	0.5%
Missing BUSCOs	8	0.6%
Total BUSCO groups searched	1367	

Recently, a chromosome-level genome assembly of the Chinese population of *T. dichotomus* was generated through a combination of ONT long read and Hi-C sequencing, as reported by Wang et al.^46^. We compared our *T. dichotomus* assembly of the Japanese population with that of the Chinese population. The dot plot indicated a high degree of congruence in the chromosomal structure (Fig. S2a), with very minor indels and inversions, which confirms the accuracy of both assemblies and also highlights the evolutionary conservation of chromosomal structures between populations. On the other hand, the whole-genome alignment of the genomes of the two populations detected a substantial number of polymorphisms at the single nucleotide resolution. Among the 584,585,878 correspondingly mapped bases, 9,457,239 bases (1.6%) were different (single nucleotide polymorphisms; SNPs) between the two genomes, and 1,308,368 indels were detected (Fig. S2b). Genetic partitioning and divergence have been reported for *T. dichotomus* according to geographical distribution, with clear morphological differences^75^. Information on the structural variations identified here will be a useful source for studying the evolution of *T. dichotomus* through comparative genomics approaches.

We assembled the mitochondrial genome separately from a paired-end library using a known partial sequence of 16S rRNA of *T. dichotomus* (GenBank accession: AB178318.1) as the seed sequence. Our de novo assembly yielded a contig of 20,217 bp. We identified 2 ribosomal RNAs, 22 tRNAs, and 13 protein-coding genes (Fig. S3). The gene repertoire and the structural arrangement showed typical features of mitochondrial genomes of insects, but the total length of the mitochondrial genome was longer than that of the model coleopteran *T. castaneum* (15.8 kb (NC_003081.2)) by 4.3 kb. The increased length is mostly due to species specific repetitive elements between the s-rRNA gene and the tRNA-GLN gene (Fig. S3).

### Genome annotation

The *T. dichotomus* genome is rich in repetitive elements, which total 305 Mb and account for 49.5% of the genome assembly (Table S2). Considering the situation that the current assembly covers 80% of the estimated genome size, it is likely that uncovered regions contain more repetitive sequences that are generally difficult to capture by Illumina-based genome assembling. We annotated the *T. dichotomu*s genome for protein-coding genes using RNA-seq data. Aiming for a comprehensive gene identification, we collected RNA samples from a wide variety of tissues of both sexes and developmental stages. We generated 16 libraries derived from 6 tissues (brain, Malpighian tubule, hindgut, fat body, testis and ovary) and from 6 time points of whole embryos from middle to late developmental stages (Table S1). The alignment data of these RNA-seq sequences mapped on the TdicSN1.0 assembly were subjected to the BRAKER2 pipeline to predict protein-coding genes. In total, 23,987 protein-coding genes are predicted in the *T. dichotomus* genome. Of these, 19,708 (82.6%) encoded proteins exhibiting sequence similarity (e-value < 1.0e−5) to proteins in the NCBI non-redundant protein database. The two most frequent top-hit species corresponded to scarab beetles, *Oryctes borbonicus*, (6312) and *O. taurus* (5126), followed by other Coleoptera species such as *T. castaneum*, *Ignelater luminosus*, and *Nicrophorus vespilloides*, which reasonably reflects the phylogenetic position of *T. dichotomus* (Fig. 1b). We used InterProScan to query the predicted coding regions for known functional domains. We identified 29,549 Pfam motifs^76^ and 34,594 PANTHER motifs^77^ in the products of 14,497 and 15,139 *T. dichotomus* gene models, respectively. We also identified 2389 proteins with secretion potential predicted by the SignalP program^78^. By integrating protein-domain based and orthology-based approaches using InterProScan and eggNOG-mapper pipelines, 581,644 Gene Ontology terms were assigned to 10,541 genes (43.9%).

### Ortholog analysis

**Figure 2:**
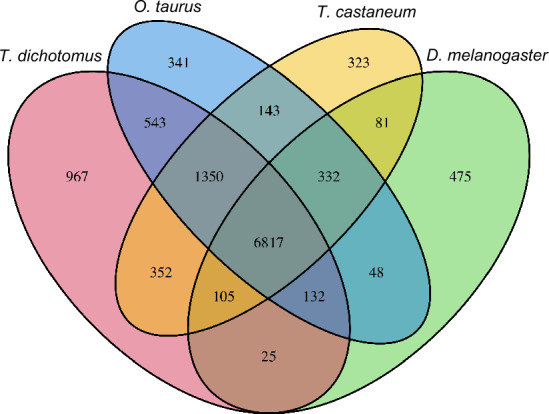
Venn diagram of shared and unique orthogroups in four insects. Orthogroups were identified by clustering of orthologous groups using OrthoFinder^60^.

To understand the gene repertoire evolution of *T. dichotomus*, we generated clusters of orthologous and paralogous gene families comparing the *T. dichotomu*s proteome with those of three other insects, *O. taurus*, *T. castaneum* and *D. melanogaster*. *O. taurus* is a dung beetle belonging to the family Scarabaeidae (Fig. 1b), and represents another model insect for the study of horn polyphenism^79^. We included *T. castaneum* as a model Coleoptera and *D. melanogaster* as a model insect for comparison as outgroups. The OrthoFinder program identified 12,034 orthogroups consisting of 56,656 genes derived from all four insects. The 18,783 T*. dichotomus* gene models were clustered into 10,291 orthogroups, leaving 5204 genes unassigned to any orthogroups (i.e., orphan genes). Among them, 1350 groups were shared within only three Coleoptera species, while 543 groups were shared within only the horned beetles, *O. taurus* and *T. dichotomus* (Fig. 2). These beetle-specific groups may account for the common characteristic traits such as the elytron of the beetle^80^ and the exaggerated horns of *T. dichotomus* and *O. taurus*. We found 967 groups, consisting of 5705 genes, that are unique to *T. dichotomus* and may account for lineage specific traits (Fig. 2).

### Transcriptome analysis

**Figure 3:**
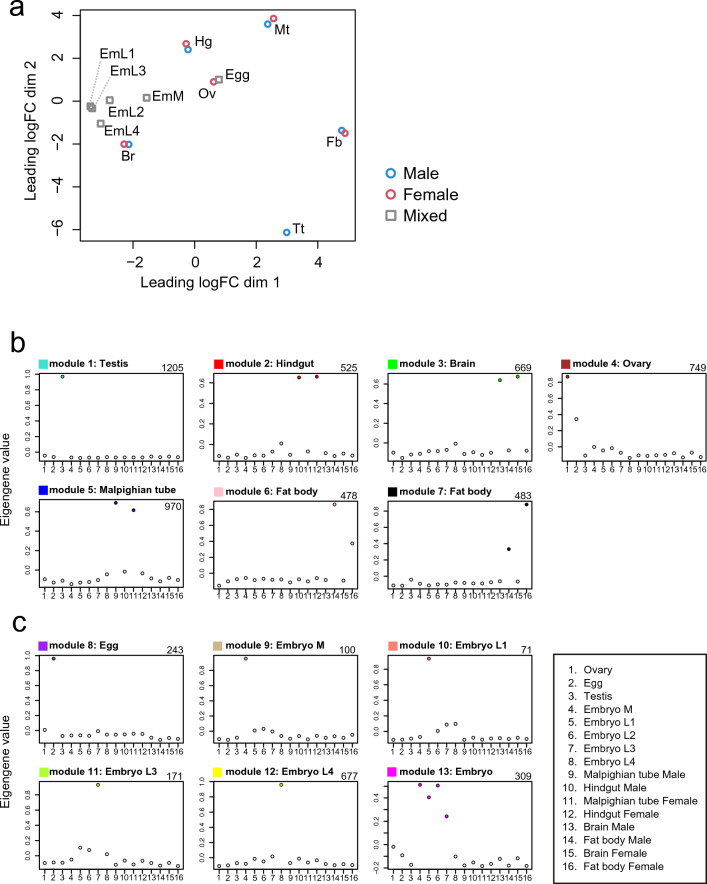
Transcriptome analysis. (**a**) MDS plot for RNA-Seq gene expression of *T. dichotomus* tissues, organs and developmental samples. Multi-dimensional scaling (MDS) plot showing relatedness between transcript expression profiles of 16 RNA-Seq libraries. Blue circles represent the expression profiles of male samples, and red circles represent those of female samples, while grey squares represent those of samples whose sex are unknown (i.e., embryos). The labels indicating the tissues and sources are defined as follows: Egg, eggs dissected out from the mature ovary; Tt, testis; Ov, ovary; EmM, middle-stage embryos at the stage of ventral appendage formation; EmL1, late stage embryo at the stage when appendages extend; EmL2, late-stage embryos at the stage when appendages are slightly more developed than EmL1; EmL3, late-embryos at a stage when the tracheal pits can be clearly observed; EmL4, late-embryos at a stage of the full-grown embryo; Mt, Malpighian tubules of third instar larva; Hg, hindgut tube of third instar larva; Fb, fat bodies of third instar larva; Br, brains of third instar larvae. See Table S1 for details of the label description. (**b**, **c**) Co-expression gene modules identified using WGCNA are shown. Each circle represents the value of the respective module’s Eigengene. Each module represents genes with highly correlated expression profiles, either in a single organ (**b**) or in a certain stage of embryogenesis (**c**). Module names and colors shown above each panel correspond to Fig. S4. The name of the organ or developmental stage in which module genes are preferentially expressed is displayed at the right of the module name in each panel. The number at the top right in each panel indicates the number of genes belonging to the module specified.

We analyzed 16 RNA-seq libraries created from a variety of tissues of both sexes and developmental stages (Table S1). We quantified the expression levels of 23,987 protein-coding genes and profiled the expression patterns across all of the sample tissues. The multidimensional scaling (MDS) plot of the 16 samples depict the transcriptome similarities among the samples (Fig. 3a). Samples derived from the same organs clustered together irrespective of sex differences. Eggs and ovaries also clustered together, as did the transcriptomes of embryos. Notably, embryonic samples were roughly ordered on the plot from earlier to later stages, which may represent the gradual transcriptional progression during embryogenesis of *T. dichotomus.*

We performed Weighted Gene Correlation Network Analysis (WGCNA) to understand the co-expression relationship between genes at a system level. WGCNA identified 13 co-expressed modules from the expression data spanning 16 samples; each module contained 71 to 1205 co-expressed genes (Figs. 3b, c, S4). Each module represents genes with highly correlated expression profiles, either in a single organ (Fig. 3b) or in a certain stage of embryogenesis (Fig. 3c). Out of 13 modules, seven represent organ-specific patterns (Figs. 3b, S4, module 1–7). Both modules 6 and 7 represent fat body preferential expression, but genes of module 6 exhibited male-biased expression while genes in the module 7 exhibited female-biased expression, suggesting sexually differentiated functions of *T. dichotomus* fat body in the sexes. Six modules represent embryonic expression patterns (Figs. 3c, S4, module 8–13). Genes contained in the module 13 exhibited a constant pattern of expression from middle to late stages.

### Horn formation genes

The horns of *T. dichotomus* provide an excellent model for investigating the evolution of novel morphological structures. Our previous research efforts to identify genes involved in horn formation in *T. dichotomus* were limited by the lack of genomic information and were confined to genes with well-known functions that were conserved across species^11–16^. However, the availability of a genome resource for *T. dichotomus* now allows us to take a comprehensive, whole-genome approach to understanding the genetic basis of horn formation, as demonstrated by the following examples.

The analysis of orthologs among four insects (*T. dichotomus, O. taurus*, *T. castaneum* and *D. melanogaster*; see above*)* revealed that 543 orthogroups were shared between two horned beetles, *O. taurus* and *T. dichotomus,* but not with the horn-less beetle *T. castaneum*. These orthologs may provide insight into the characteristic trait of horns found in both species. While functions of most of these genes are currently unknown, those annotated as transcription factors are of particular interest as they may play a role in regulating the gene regulatory network responsible for horn formation (Table S3).

It is widely acknowledged that gene duplication plays a significant role in the acquisition of evolutionarily novel traits^81^. In this study, we investigated the potential role of gene duplication in *T. dichotomus*. Specifically, we focused on 17 genes that have been previously linked to horn formation in this species (*abrupt*, *Bar-H1*, *CyclinE*, *dachshund*, *doublesex*, *fat*, *Insulin-like receptor, intersex*, *Notch*, *Optix, pannier*, *Retinal Homeobox*, *Sex combs reduced*, *Sox14*, *Sox21b*, *Sp8* and *Tbx20*)^11–16^. We found that all of these genes were single-copy with no evidence of duplication. This suggests that the involvement of these 17 genes in horn formation may have resulted from the co-option of existing genes, rather than gene duplication followed by neo-functionalization.

**Figure 4:**
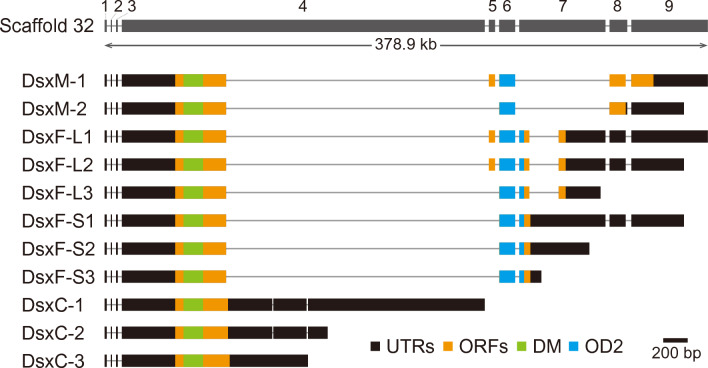
Genome organization of *dsx* by alignment with genome sequences. The upper gray box indicates the *dsx* exons localized at scaffold 32. Information on the *T. dichotomus dsx* isoforms was obtained from in GenBank sequence data (accession numbers AB744665–AB744675). Black boxes indicate untranslated regions (UTRs), Orange boxes indicate open reading frames (ORFs), and Green and Blue boxes indicate the functional domains, DM (Doublesex/Mab-3 DNA-binding; OD1) and OD2 (Oligomerization domain 2) domains. Exons 1, 2 and 3 were difficult to align due to their short sequence, thus the closest conserved region from Exon 4 was selected. It should be noted that the locus of Exons 1, 2 and 3 is uncertain as a result. The scale bar, representing 200 bp, applies only to the exon region, while the intron regions are arbitrary and not to scale. The exon number is indicated above each exon.

The availability of genome sequence enables us to gain a deeper understanding of the genomic organization of genes. In *T. dichotomus*, the sex differentiation gene *doublesex* (*dsx*) plays a crucial role in horn formation^11^, and sex-specific splicing has been identified as a key mechanism for regulating this process^12^. There are various splicing variants in the *T. dichotomus dsx* gene (such as *dsxFL-1*, *dsxFL-2*, *dsxFL-3*, *dsxFS-1*, *dsxFS-2*, *dsxFS-3*, *dsxM-1*, *dsxM-2*, *dsxC-1*, *dsxC-2* and *dsxC-3*)^11^. *DsxFLs* (female-specific isoforms) and *dsxMs* (male-specific isoforms) each have roles in repressing and promoting horn formation, respectively. Prior to the availability of the genome sequence, the exon–intron structure of the *dsx* gene was predicted based on cDNA sequences alone^11^. However, the alignment of transcripts and the genome reference in the *dsx* locus has allowed for a more precise determination of the gene structure. We discovered that the *dsx* locus spans 379 kb on Scaffold 32 and comprises 9 exons (Fig. 4). In addition, we found that the regulation of alternative splicing of *dsx* genes was more complex than previously inferred from cDNA sequence analysis. For example, the Exon 8 of the *dsxM2* gene is 10 bases longer than that of *dsxM1,* leading to an additional stop codon and a distinct protein being translated. Furthermore, non-sex-specific *dsx* isoforms (*dsxC*) are not spliced at Exon 4, a finding that has been observed in other insect species including *Apis mellifera*, *O. taurus*, and *Gnatocerus cornutus*, as well as in *T. dichotomus*^82–84^. The presence of *dsxC* in these insects may have been a result of intron retention as in *A. mellifera* and *T. dichotomus*. We also found that the only difference between *dsxFLs* and *dsxMs*, which are involved in sex-specific horn formation, lies in the splicing pattern of Exon 7. This finding suggests that splicing of Exon 7 is crucial and that this splicing pattern determines the presence or absence of horns.

## Conclusions

We report the assembly and annotation of the nuclear and mitochondrial genomes of the Japanese rhinoceros beetle *T. dichotomus septentrionalis.* The high level of completeness and the extensive annotation of this genome, in conjunction with transcriptome data collected from diverse tissues and developmental stages, make this draft assembly an excellent resource for further functional and evolutionary analyses in this emerging model insect. In fact, we used genomic information to determine the precise exon–intron structure of the *dsx* gene in *T. dichotomus,* and propose that the splicing pattern of exon 7 is critical in determining the presence or absence of horns. Furthermore, the availability of the genome sequence will expedite the use of functional assays using RNAi and genome editing techniques. These are key tools for experimental investigations of the evolutionary origin and genetic regulation of the exaggerated male horn, and the molecular mechanisms underlying the strong sexual dimorphism in its expression. Other research areas such as ethology, biomimetics and drug discovery will benefit from the continuing advancement of genomic resources. Finally, given that *T. dichotomus* is one of the most popular insects in Japan, we hope that the genomic resources will facilitate the development of effective population genomic tools to monitor and protect wild populations, which would lead to the establishment of the conservation genomics of the Japanese rhinoceros beetle*.* These genomic data and a genome browser are available at http://www.insect.nibb.info/trydi/.

## Supplementary Information

Supplementary Information 1.

Supplementary Information 2.

## Data Availability

Data from whole-genome sequencing and transcriptome sequencing have been deposited in the DDBJ database under BioProject accession PRJDB12657. The analyzed data including genome assembly, gene prediction, annotation, and gene expression are available through FigShare (10.6084/m9.figshare.c.5737754). The genome browser is available at http://www.insect.nibb.info/trydi/.
