# Supplementary material for: The draft genome sequence of the Japanese rhinoceros beetle Trypoxylus dichotomus septentrionalis towards an understanding of horn formation
Source: Sci Rep. 2023 May 30;13:8735. doi: 10.1038/s41598-023-35246-w (PMC10229555; doi:10.1038/s41598-023-35246-w)
Supplement: Supplementary file 1 — Supplementary Information 1. [file 41598_2023_35246_MOESM1_ESM.pdf]

Fig. S1

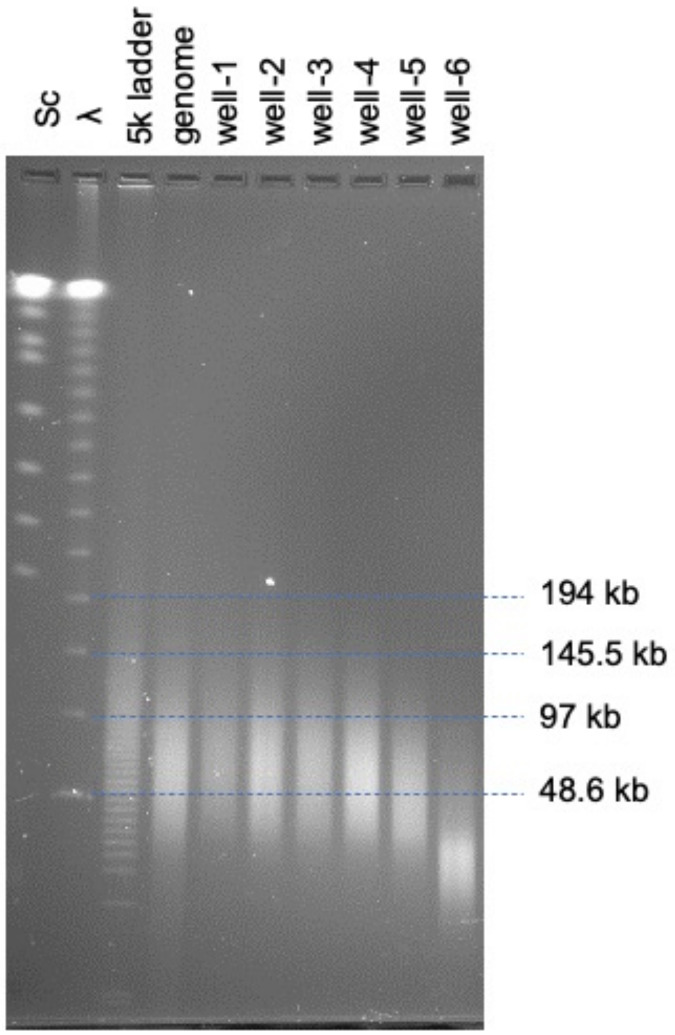

Figure S1. Size distribution of purified high-molecular weight (HMW) genome DNA and size-fractioned DNA assessed by pulsed-field gel electrophoresis (PFGE). The PFGE analysis revealed that the HMW DNA had a mean size of approximately 50 - 80 kbp. The fraction labeled with well-1, which retained long DNA while effectively removing shorter DNA fragments (< ~10 kbp) through size fractionation using the SAGE HLS system, was selected for the linked-read library preparation. Sc: Saccharomyces cerevisiae genome, λ: lambda ladder, 5k ladder: 5 kbp ladder. genome: purified high-molecular weight genome DNA. well-1 to -6 : DNA size-binned with SAGE HLS system. well-1 represents the longest fraction and well-6 represents the shortest one.

Fig. S2

a

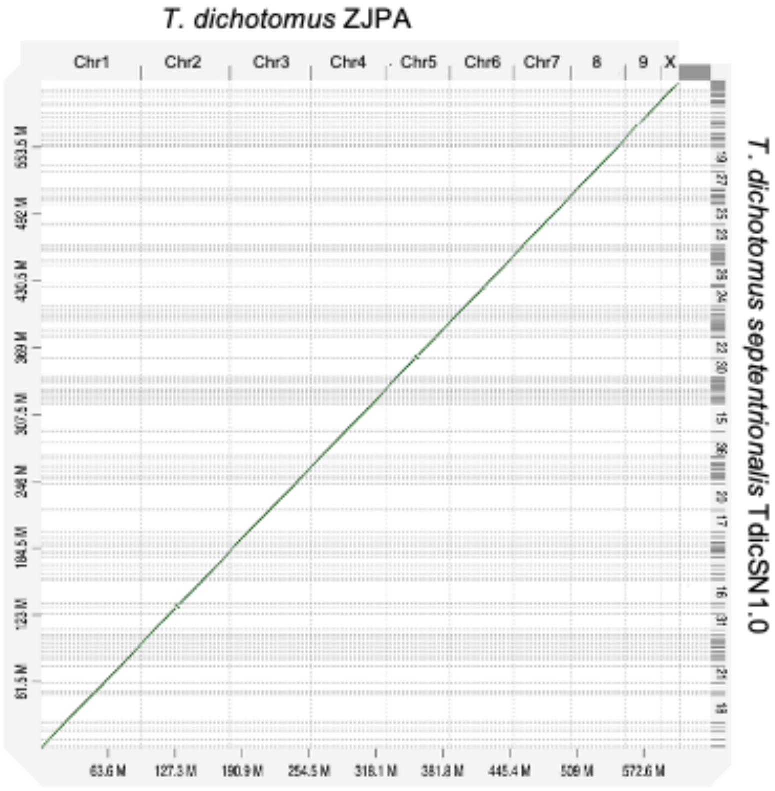

b

Number of mapped sequences	15,144
Number of bases in mapped sequences	614,675,032
Number of mapped bases	584,585,878
Number of substitutions (SNPs)	9,457,239
Number of insertions < 50 bp	585,443
Number of insertions (50-100 bp)	8,850
Number of insertions (100-300 bp)	6,283
Number of insertions in (300-400 bp)	981
Number of insertions (400 - 1000 bp)	2,487
Number of insertions > 1k bp	3,598
Number of deletions < 50 bp	636,319
Number of deletions (50 - 100 bp)	23,942
Number of deletions in (100 - 300 bp)	19,562
Number of deletions in (300 - 400 bp)	3,244
Number of deletions in (400 - 1000)	7,485
Number of deletions in > 1k bp	10,174

Figure S2. Whole genome comparison of *T. dichotomus* between two isolates. We compared the genome assemblies of *T. dichotomus* genome assembly TdicSN1.0 derived from a Japan population and the chromosome-level assembly of the *T. dichotomus* ZJPA isolate derived from a China population[46]. (a) A dot plot representation of the two assemblies. (b) The structural variations identified between the two assemblies.

Fig.S3

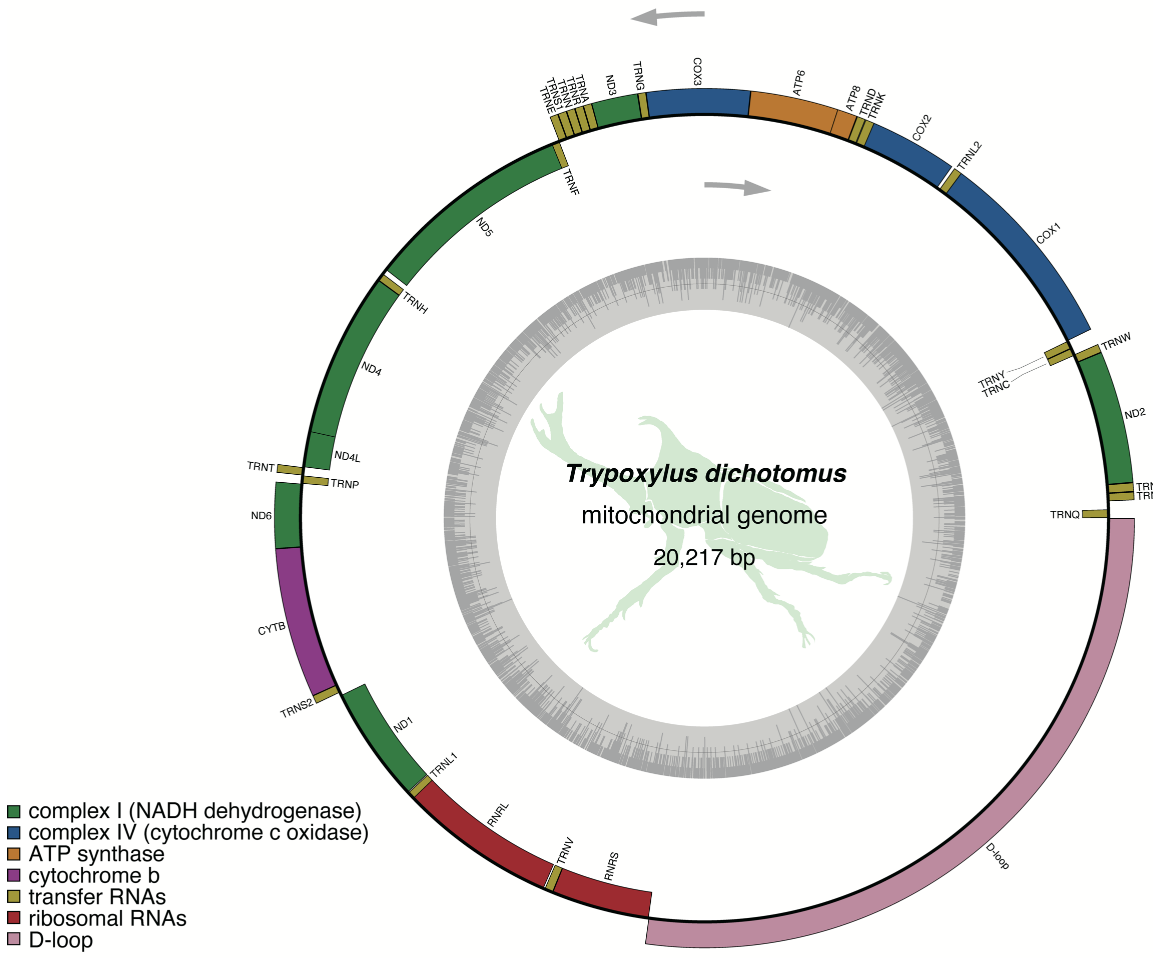

Figure S3. Schematic of the mitochondrial genome of *T. dichotomus.* Gray arrows indicate the transcription direction of DNA strands. The colors of the genes indicate different functional categories. The details are shown in the legend of the figure. The GC content of the mitochondrial genome is indicated by the inner gray circle. The circle inside the GC content graph shows the 50% threshold.

Fig.S4

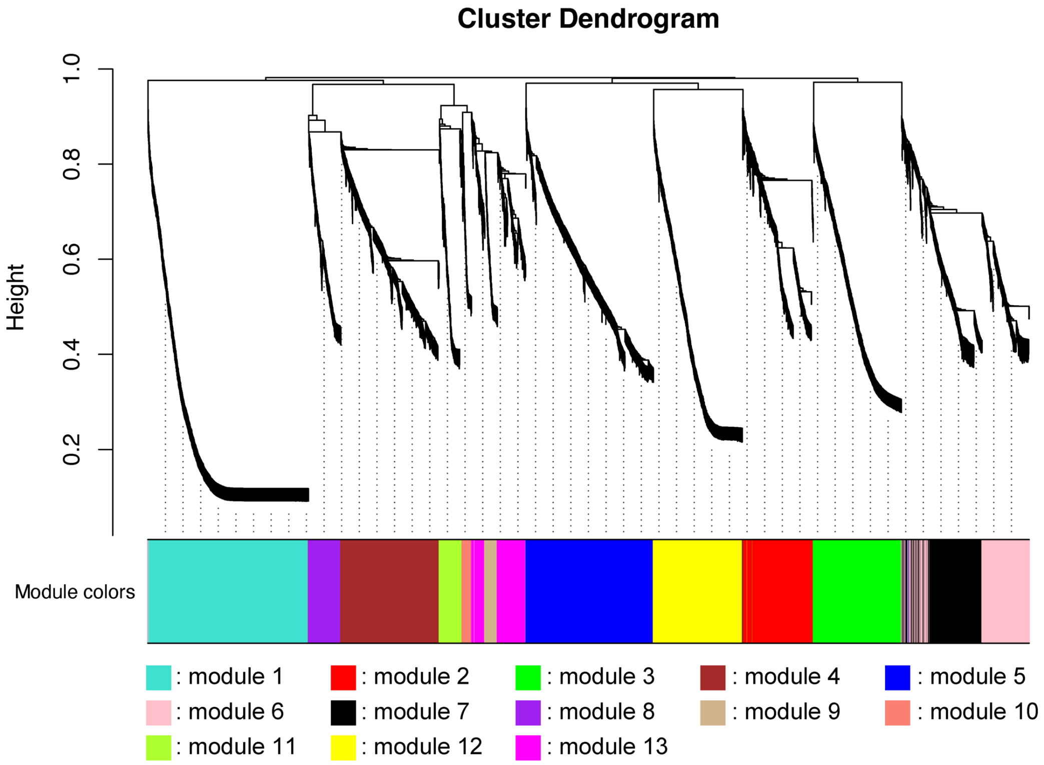

Figure S4. Gene co-expression analysis of the *T. dichotomus* transcriptome. Hierarchical cluster tree of the *T. dichotomus* genes showing co-expression modules identified using WGCNA. Modules correspond to branches and are labeled by colors as indicated by the color band underneath the tree. In total, 13 co-expression modules were identified from the expression data of 16 samples.
